# The Gustin (CA6) Gene Polymorphism, rs2274333 (A/G), as a Mechanistic Link between PROP Tasting and Fungiform Taste Papilla Density and Maintenance

**DOI:** 10.1371/journal.pone.0074151

**Published:** 2013-09-09

**Authors:** Melania Melis, Elena Atzori, Stefano Cabras, Andrea Zonza, Carla Calò, Patrizia Muroni, Mariella Nieddu, Alessandra Padiglia, Valeria Sogos, Beverly J. Tepper, Iole Tomassini Barbarossa

**Affiliations:** 1 Department of Biomedical Sciences, University of Cagliari, Monserrato, Italy; 2 Department of Life and Environment Sciences, University of Cagliari, Monserrato, Italy; 3 Department of Food Science, School of Environmental and Biological Sciences, Rutgers University, New Brunswick, New Jersey, United States of America; German Institute of Human Nutrition Potsdam-Rehbruecke, Germany

## Abstract

Taste sensitivity to PROP varies greatly among individuals and is associated with polymorphisms in the bitter receptor gene *TAS2R38*, and with differences in fungiform papilla density on the anterior tongue surface. Recently we showed that the PROP non-taster phenotype is strongly associated with the G variant of polymorphism rs2274333 (A/G) of the gene that controls the salivary trophic factor, gustin. The aims of this study were 1) to investigate the role of gustin gene polymorphism rs2274333 (A/G), in PROP sensitivity and fungiform papilla density and morphology, and 2) to investigate the effect of this gustin gene polymorphism on cell proliferation and metabolic activity. Sixty-four subjects were genotyped for both genes by PCR techniques, their PROP sensitivity was assessed by scaling and threshold methods, and their fungiform papilla density, diameter and morphology were determined. *In vitro* experiments examined cell proliferation and metabolic activity, following treatment with saliva of individuals with and without the gustin gene mutation, and with isolated protein, in the two iso-forms. Gustin and *TAS2R38* genotypes were associated with PROP threshold (*p*=0.0001 and *p*=0.0042), but bitterness intensity was mostly determined by *TAS2R38* genotypes (*p*<0.000001). Fungiform papillae densities were associated with both genotypes (*p*<0.014) (with a stronger effect for gustin; *p*=0.0006), but papilla morphology was a function of gustin alone (*p*<0.0012). Treatment of isolated cells with saliva from individuals with the AA form of gustin or direct application of the active iso-form of gustin protein increased cell proliferation and metabolic activity (*p*<0.0135). These novel findings suggest that the *rs2274333* polymorphism of the gustin gene affects PROP sensitivity by acting on fungiform papilla development and maintenance, and could provide the first mechanistic explanation for why PROP super-tasters are more responsive to a broad range of oral stimuli.

## Introduction

Individual variability in sensitivity to the bitter taste of phenythiocarbamide was first recognized by Fox more than eight decades ago [1]. Since that time, steady progress has been made in elucidating the psychophysical features [2–5], population genetics [6,7] and molecular basis of this trait [8,9]. PTC/PROP tasting has also gained considerable attention as an oral marker for food preferences and eating habits that ultimately impacts nutritional status and health [10]. This role is based on data showing that the PROP phenotype associates with variation in perception and preference for fat [11–13], energy intake and body weight [14,15], selection of fruits and vegetables [16–18], plasma antioxidant status [19] and the risk of colon cancer [20–22]. This involvement remains controversial since some studies have failed to show the expected associations between PTC/PROP status and health outcomes [23–25]. These controversies could also be explained by confounding factors (such as cognitive control of eating behavior or the endocannabinoid system) that may play a prominent role in determining these associations [26,27].

The bitterness of PTC /PROP is due to the presence of the N–C=S group within these molecules. The human gene that expresses receptors that bind this chemical group is known as *TAS2R38*. Individuals can be divided into three taster groups (non-taster, medium taster and super-taster) based on behavioral testing assessing their PTC/PROP sensitivity. The percentage of non-taster individuals greatly varies among populations: from less than 7% to more than 40% [28]. There are two classes of screening methods: threshold determinations and suprathreshold measures that address stimulus detection and responsiveness at higher concentrations, respectively [2,10,13,14,29–36].

Allelic diversity in the *TAS2R38* bitter receptor gene is primarily responsible for PROP tasting [8,9]. Three polymorphic sites in the *TAS2R38* sequence, result in amino acid substitutions at positions Pro49Ala, Ala262Val, and Val296Ile, giving rise to two common haplotypes: PAV, the dominant (taster) variant and AVI, the recessive (non-taster) one. PROP-taster individuals possess the PAV/PAV or PAV/AVI diplotype, whereas non-tasters are homozygous for the recessive haplotype (AVI/AVI). Rare haplotypes (AAV, AAI, PVI, and PAI) have also been observed [6]. *In vitro* experiments [9] and receptor modelling [37,38] suggest that the PAV variant defines the active binding site of the receptor.

*TAS2R38* is reported to account for majority (50-85%) of the variation in the phenotype [8,9], but a variety of observations suggest that other genes [39,40] may also be involved. On the other hand, a recent genome-wide association study revealed that only loci within the *TAS2R38* gene were associated with the perception of PROP [5]. This latter finding is consistent with the idea that the TAS2R38 receptor is specific for thiourea substances, and is not activated by bitter compounds lacking the thiourea group [41,42]. Nevertheless, recent data suggest that salivary proteins may complement the direct effects of DNA sequence variation in *TAS2R38* on PROP tasting, further refining bitterness perception. Specifically, Cabras et al. [43] showed that PROP super-tasting was associated with higher salivary levels of Ps-1 and II-2 peptides belonging to the basic proline-rich protein (bPRP) family of peptides, and that oral supplementation with Ps-1 peptide enhanced the bitterness of PROP [44]. These data are consistent with the role of bPRPs as modifiers of taste and astringent molecules [45–47].

Our laboratory has also been studying the role of the zinc dependent salivary protein, gustin (also known as carbonic anhydrase VI (CA6)), in PROP tasting [48,49]. Gustin/CA6 is a 42 kDa protein secreted by the parotid, submandibular and von Ebner glands [50–52]. Gustin is considered a trophic factor that promotes growth and development of taste buds since disruptions in this protein are known to decrease taste function [53]. Padiglia et al. [48] showed that the rs2274333 (A/G) polymorphism of the gustin gene results in an amino acid substitution at position Ser90Gly in the peptide, leading to a structural modification of the gustin active site, reduced zinc binding, and the accumulation of zinc ions in saliva. This gustin polymorphism is also strongly associated with PROP tasting [48] such that PROP super-tasters more frequently carried the AA genotype of gustin and expressed the native form of the protein, whereas PROP non-tasters more frequently carried the GG genotype and expressed the less functional form [49]. PROP super-tasters have a greater density of fungiform taste papillae on the anterior surface of the tongue [2,34,54–56]. Considering gustin’s role in taste bud development and the close association between the rs2274333 polymorphism of gustin and PROP tasting, it is plausible that the relationship between papillae density and PROP status is mediated by gustin. To date, no studies have examined the effects of gustin on taste papilla morphology and physiology, particularly with respect to PROP taster status.

The objectives of this study were to investigate the effect of gustin gene polymorphism rs2274333 (A/G) and *TAS2R38* polymorphisms on PROP sensitivity and fungiform papillae density and morphology in a genetically homogeneous cohort. In addition, *in vitro* experiments, examined 1) the effect of treatment with saliva collected from individuals with genotype AA and GG of polymorphism rs2274333 on cell development and metabolic activity, and 2) the effect of treatment with isolated gustin, in the two iso-forms resulting from this polymorphism, on cell metabolic activity.

## Materials and Methods

### Ethical statement

All subjects was verbally informed about the procedure and the aim of the study. They reviewed and signed an informed consent form. The study was conformed to the standards set by the latest revision of Declaration of Helsinki and the procedures have been approved by the Ethical Committee of the University Hospital of Cagliari, Italy.

### Subjects

Sixty-three non-smoking Caucasian healthy, young subjects (22 males, 42 females, age 25 ± 3 y) from Sardinia, Italy were recruited at the local University. They had a normal body mass index (BMI) ranging from 18.6 to 25.3 kg/m^2^ and showed no variation of body weight larger than 5 kg over the previous 3 months. None were following a prescribed diet or taking medications that might interfere with taste perception. Subjects neither had food allergies, nor scored high on eating behaviour scales (assessed by the Three-Factor Eating Questionnaire [57]). Thresholds for the 4 basic tastes (sweet, sour, salty, bitter) were evaluated in all subjects in order to rule out any gustatory impairment.

### PROP taste sensitivity assessments

The PROP phenotype of each subject was assessed by both threshold and suprathreshold measures. PROP (Sigma-Aldrich, Milan, Italy) thresholds were determined using a variation of the ascending-concentration, 3-alternative forced-choice (3-AFC) procedure [58]. PROP solutions in spring water ranged from 0.00001 to 32 mM in quarter-log steps.

Taste intensity ratings for a single suprathreshold PROP (3.2 mM) solution [49] were collected using the Labeled Magnitude Scale (LMS) [59] in which subjects placed a mark on the scale corresponding to his/her perception of the stimulus. The LMS scale gave subjects the freedom to rate the PROP bitterness relatively to the “strongest imaginable” oral stimulus they had ever experienced in their life.

For both methods, the solutions were prepared the day before each session and stored in the refrigerator until 1 h before testing. The stimuli were presented at room temperature as 10 ml samples.

### Molecular analysis

Subjects were genotyped for polymorphism rs2274333 (A/G) of the gustin (CA6) gene that consists of a substitution of amino acid Ser90Gly. They were also genotyped for three single nucleotide polymorphisms (SNPs) at base pairs 145 (C/G), 785 (C/T), and 886 (G/A) of the *TAS2R38* locus (through the manuscript the name of the gene is identified in italics, while its corresponding encoded protein by plain text). The *TAS2R38* SNPs give rise to 3 non-synonymous coding exchanges: proline to alanine at residue 49; alanine to valine at residue 262; and valine to isoleucine at residue 296. These substitutions result in two major haplotypes (PAV and AVI) and three rare (AAI, PVI and AAV). The DNA was extracted from saliva samples using the Invitrogen Charge Switch Forensic DNA Purification kit (Invitrogen, Carlsbad, CA, USA) according to the manufacturer’s instructions. Purified DNA concentration was estimated by measurements of OD260. PCR techniques were employed to amplify the gustin gene region including rs2274333 polymorphism, and the two short region of the *TAS2R38* gene including the three polymorphisms of interest.

To genotype gustin gene polymorphism rs2274333, a fragment of 253 bp was amplified with forward 5'TGACCCCTCTGTGTTCACCT3' and reverse 5'GTGACTATGGGGTTCAAAGG3' primers. The reaction mixtures (25 µL) contained 250 ng DNA, 10 pmol of each primer, 1.5 mM MgCl_2_, 100 mM Tris-HCl at pH 8.3, 50 mM KCl, 200 µM of dNTP mix, and 1.5 units of Hot Master Taq Eppendorf. Thermal cycles of amplification were carried out in a Personal Eppendorf Master cycler (Eppendorf, Germany). The amplification protocol included an initial denaturation at 95°C for 5 min, followed by 35 cycles of denaturation at 95°C for 30 s, annealing at 54°C for 30 s, and then extension at 72°C for 30 s. A final extension was carried out at 72°C for 5 min. Amplified samples were digested with HaeIII enzyme at 37°C for 4 hours. The digested fragments were electrophoresed on 2% agarose gel and stained with ethidium bromide.

To determine *TAS2R38* haplotypes, PCR amplification followed by restriction analysis using *Hae*III for SNP detection at the 145 nucleotide position, and direct sequencing (using forward and reverse primers) for SNPs identification at the 785 and 886 nucleotide position. The following primer set was used to amplify a fragment of 221 bp including the first of three SNPs : F5’-CCTTCGTTTTCTTGGTGAATTTTTGGGATGTAGTGAAGAGGC**G**G-3’ R 5'-AGGTTGGCTTGGTTTGCAATCATC-3'. The forward primer binds within the *TAS2R38* gene, from nucleotides 101–144. There is a single mismatch at position 143, where the primer has a G (underlined in bold) and the gene has an A. This mismatch is crucial to the PCR experiment, because the A nucleotide in the *TAS2R38* gene sequence, is replaced by a G in each of the amplified products. This creates the first G of the *Hae*III recognition sequence GGCC, allowing the amplified taster allele to be cut. The amplified non taster allele reads GGGC and is not cut. The PCR reaction mixtures (25 µL) contained 250 ng DNA, 10 pmol of each primer, 1.5 mM MgCl_2_, 100 mM Tris-HCl at pH 8.3, 50 mM KCl, 200 µM of dNTP mix, and 1.5 units of Hot Master Taq Eppendorf. Thermal cycles of amplification were carried out in a Personal Eppendorf Master cycler (Eppendorf, Germany). The amplification protocol consisted of initial denaturation at 95°C for 5 min, followed by 35 cycles of denaturation at 95°C for 30 s, annealing at 64°C for 45 s, and then extension at 72°C for 45 s. For the analysis of the polymorphism G/C at position 143, a 3 µl aliquot of PCR products was mixed with a 17 µl solution containing 2 µl 10 × NE Buffer (50mM NaCl, 10mM Tris–HCl, 10mM MgCl_2_, 1mM dithiothreitol, pH 7.9), 0.2 μ HaeIII (10 000 U ml^-1^; Sigma-Aldrich, St Louis, MO), and 14.8 µl sterile deionized H_2_O. The solution was incubated at 37°C for 4 h. The digest was mixed with 5 ml of loading buffer and electrophoresed on a 10% vertical polyacrylamide gel. The DNA bands were evidenced by ethidium bromide staining. The PCR 100 bp Low Ladder DNA was used as Mr markers (Sigma-Aldrich). Polymorphisms at the 785 and 886 nucleotide position were identified by a single PCR reaction using the sense primer 5’-TCGTGACCCCAGCCTGGAGG-3’ and the antisense primer 5’-GCACAGTGTCCGGGAATCTGCC-3’ delimiting a 298 bp fragment. The PCR reaction mixtures (25 µL) contained 250 ng DNA, 10 pmol of each primer, 1.5 mM MgCl_2_, 100 mM Tris-HCl at pH 8.3, 50 mM KCl, 200 µM of dNTP mix, and 1.5 units of Hot Master Taq Eppendorf. Thermal cycles of amplification were carried out in a Personal Eppendorf Master cycler (Eppendorf, Germany). The amplification protocol consisted of initial denaturation at 95°C for 5 min, followed by 30 cycles of denaturation at 95°C for 30 s, annealing at 60°C for 30 s, and then extension at 72°C for 30 s. PCR products were sequenced with an ABI Prism automated sequencer. Nucleotide and deduced amino acid sequence analyses were performed with the OMIGA version 2.0 software (Oxford Molecular, Madison, WI).

### Fungiform papillae identification and measurements

The method to identify fungiform papillae was similar to that developed by Shahbake et al. [56] and is briefly described as follows. The tip of the anterior tongue surface was dried with a filter paper and stained by placing (for 3 s) a piece of filter paper (circle 6 mm in diameter) that contained a blue food dye (E133, Modecor Italiana, Italy) at the left side of the midline. Photographic images of the stained area were taken using a Canon EOS D400 (10 megapixels) camera with lens EFS 55-250 mm. Three to ten photographs were taken of each subject, and the best image was analyzed. The digital images were downloaded to a computer and were analyzed using a “zoom” option in the Adobe Photoshop 7.0 program. The fungiform papillae were identified from the digital images by their mushroom-shape, they were readily distinguished from filiform papillae by their very light staining with the food dye compared to the latter papillae which stained dark [60].

The number of papillae in the stained area was counted for each subject, and the density in (1 cm^2^) was calculated. The diameter of each papilla was measured in 4 dimensions (at 0, 45, 90 and 135°) and the standard deviation (SD) was calculated. This procedure was repeated for all papillae in a counting area. A fungiform papilla was considered distorted when the SD was ≥ 0.088. This value corresponded to 2 SDs. The grand mean of diameters, the mean of SDs, and the percentage of distorted fungiform papillae were determined for each subject. Papillae were separately evaluated by three trained observers who were blind to the PROP status of the subjects. The final measurements were based on the consensus assessment of all observers.

### Experimental procedure

Subject testing was carried out in three visits on different days separated by a 1-month period. Subjects were requested to abstain from eating, drinking and using oral care products or chewing gums for at least 8 h prior to testing. They had to be in the test room 15 min before the beginning of the session (9.00 AM) in order to adapt to the constant environmental conditions (23-24°C; 40-50% relative humidity). In the first visit, a 3 ml sample of whole saliva was collected from each subject, into an acid-washed polypropylene test tube by means a soft plastic aspirator. Samples were stored at -80°C until molecular analyses were completed as described above. After 15 min, subjects rinsed their mouth with distilled water, then the tongue was dried and stained as described above, and photographs of the tip of the tongue were recorded.

Taste assessments were carried out in the 2^nd^ and 3^rd^ visits. In women, visits were scheduled around the sixth day of the menstrual cycle to avoid taste sensitivity changes due to the estrogen phase [61]. In the second visit, after rinsing the mouth with spring water, subjects were instructed to swish the entire contents of one cup (10 mL of PROP 3.2 mM) in their mouth for 10 s and then to spit it out. After tasting, the subjects evaluated bitterness intensity of the solution using the LMS. PROP thresholds were determined for each subject at the third visit. All rinsed their mouth with spring water before the experimental session. They were presented with 3 cups positioned in a random order, one with a given PROP concentration and two containing spring water. They were instructed to swish the entire contents of one cup in their mouth for 5 s and then to spit it out. Before moving onto the next cup, they rinsed their mouth with spring water. After tasting all 3 samples, they were asked to choose which one was different from the other two samples. The detection threshold was designated as the lowest concentration at which the subject correctly identified the target stimulus on three consecutive trials. The inter-stimulus interval as well as inter-trial interval was set at 60 s.

### In vitro experiments

Two cell-based experiments were conducted. The first experiment tested the effects of treatment with saliva collected from individuals with genotype AA and GG of polymorphism *rs2274333* on cell proliferation and metabolic activity. The second one tested the effects of treatment with the two gustin iso-forms isolated from saliva of donors homozygous for AA and GG, on cell metabolic activity.

#### Cell cultures

A fetal goat tongue-derived epithelial cell line (ZZ-R 127) supplied by the Collection of Cell Lines in Veterinary Medicine of the Friedrich Loeffler Institute was used [62]. Cells were cultured in Dulbecco’s Modified Eagle’s Medium (DMEM, Gibco, USA) plus 10% (v/v) fetal calf serum (FCS, Gibco) at 37°C in a humidified atmosphere of 5% CO_2_. Cells were plated in 24-well plates at a density of 8x10^4^ cells/well. After 24 h, cells in DMEM plus 10% FCS were treated for 72 h with 10% saliva from donors (or gustin iso-forms) depending on the experimental conditions.

#### Effects of saliva on growth and metabolic activity

For the first experiment, saliva was collected from a total of 24 subjects; 12 subjects with genotype AA at the gustin locus (*TAS2R38* genotypes were as follows: 8 heterozygous and 4 PAV homozygous) and 12 subjects with genotype GG at the gustin locus (*TAS2R38* genotypes were: 6 AVI homozygous, 4 heterozygous and 2 PAV homozygous). Saliva was collected on the same day as the *in vitro* experiments, and centrifuged at 12,000 RPM for 10 minutes. The supernatant was filtered with a sterile 0.22-µm pore filter, and then added to the cell cultures, as described below. Gustin protein was still present in filtered supernatants, as demonstrated by immunoblot experiments (data not shown). Three experimental treatments were used: (1) saliva from subjects with genotype AA; (2) saliva from subjects with genotype GG; and (3) control (DMEM plus 10% FCS alone). Saliva from each subject was assayed separately. After 72 h treatment, cells were trypsinized and counted with a hemocytometer under inverted microscope.

Cell metabolic activity was determined by the resazurin system (Tox-8 assay kit, Sigma, USA) in which metabolically active cells convert resazurin into a fluorescent dye, resorufin, by the intracellular reduction enzymes. This assay represents a simple, accurate and reproducible tool for measuring the metabolic activity of living cells [63]. After 72h treatment with saliva, resazurin dye solution was added to cells in an amount equal to 10% of the culture medium volume (100 µl/well) and cells were cultured for a further 4 h. Fluorescence of converted dye was measured using a fluorescent microplate reader (VICTOR X Multilabel Plate Readers, PerkinElmer) at a wavelength of 590 nm using an excitation wavelength of 560 nm.

Mean values of cell number and fluorescence emission after treatments with saliva of subjects with genotype AA (n=12) and genotype GG (n=12) were calculated and are presented graphically.

#### Effects of gustin iso-forms on metabolic activity

In the second experiment, cells were treated with isolated gustin in the two iso-forms resulting from the polymorphism *rs2274333* (A/G). Saliva was collected from one super-taster donor homozygous for the AA form of gustin (*rs2274333*) and from one non-taster donor homozygous for the GG form (both heterozygous for *TAS2R38*), and used to purify the two iso-forms of carbonic anhydrase VI. The preparation of saliva samples and all purification steps were conducted using the method of Murakami and Sly [64]. The same experimental procedure was used for the purification of each iso-form. Volunteers expectorated in a frozen bottle containing 2 ml of 0.2 M benzamidine (Sigma-Aldrich, St. Louis, MO) in 0.1 M Tris-SO4, and 0.2 M sodium sulfate, at pH 8.7. Saliva samples were collected after lunch, because food intake enhances the secretion of saliva from the parotid glands which are the primary site for gustin protein production [65]. Samples of whole saliva were collected from each subject, after stimulation with citric acid. This produced large amounts (~40 mL) per collection. The collection procedure was repeated in different days until a pooled sample of 250 ml of saliva for each genotype was obtained. Samples were stored at -80^c^ then thawed and centrifuged (16,000 x g, 15 min) to remove foreign material. The supernatant was diluted to 1 liter with 0.1 M Tris-SO4, and sodium sulfate 0.2 M at pH 8.7.

The purification of carbonic anhydrase VI was carried out through the use of affinity chromatography, preparing the column matrix as reported by Khalifah et al. [66]. Specifically, carboxy methyl Bio-Gel A (Bio-Rad Laboratories, Richmond, CA) was linked to the sulfonamide inhibitor p-aminomethylbenzenesulfonamide (Gallade Chemical; Newark, CA). EDAC [1-(3-dimethylamionpropyl)-3-ethyl carbodiimide hydrochloride] obtained from Sigma-Aldrich (St. Louis, MO), was used to activate the column matrix carboxyl groups. The purified fractions containing the carbonic anhydrase VI were collected based on spectrophotometric absorbance values at 280 nm. Then, as reported by Murakami and Sly [64], fractions containing the protein were applied to a diethylaminoethyl -sephacel (Sigma-Aldrich, St. Louis, MO) ion-exchange column. The concentration of purified protein was quantified by the method of Lowry et al. [67] using bovine serum albumin as a standard, and its purity was determined by SDS-PAGE (sodium dodecyl sulfate-polyacrylamide gel electrophoresis). SDS-PAGE (12% acrylamide) was performed according to Laemmli [68]. Sigma, Marker product code C 4236 (Sigma-Aldrich, St. Louis, MO) with range 8-210 kDA was employed as a standard in electrophoresis. The gel was stained with Coomassie Brilliant Blue R-250 (Sigma-Aldrich, St. Louis, MO), using the typical Coomassie staining procedure [69]. The yield of the purification was approximately 1 mg of protein starting from 250 ml of whole saliva.

The mean concentration of gustin in human saliva is about 5 ± 0.2 µg/ml [70]. Since gustin binds an ion of Zn with a stoichiometry of 1:1 [50], we used a protein concentration of 8 µg/ml corresponding to 0.2 nmoles, and 0.2 nmoles of added Zn. Four experimental treatments were used: (1) gustin Ser90 + Zn; (2) gustinGly90 + Zn; (3) control (DMEM plus 10% FCS alone); and (4) control + Zn. The Tox8 assay (previously described) was used to obtain fluorescence emissions using the same procedures as the saliva experiment. Since we were able to obtain a large amount of isolated protein, each treatment was repeated 33 times (to maximize the reliability of the assay) and the mean values of the replicates are presented graphically.

### Statistical analyses

Hardy Weinberg equilibrium for the *TAS2R38* gene and polymorphism 2274333 (A/G) of the gustin gene was verified through the Markov Chain test (Genepop software version 4.0; http://kimura.univ-montp2fr/~rousset/Genepop.htm). Linkage disequilibrium (LD) between the two loci was verified by the Markov Chain algorithm (Genepop software version 4.0.5.3; http://kimura.univ-montp2fr/~rousset/Genepop.htm). We stratified our sample based on *TAS2R38* and gustin genotypes, and tested both the additive and dominant models for the PAV and A variants, respectively, with the Chi square test to show the two genes are independent.

Main effects ANOVA was used to examine the effects of the *TAS2R38* gene and polymorphisms *2274333* (A/G) of the gustin gene on PROP threshold, bitterness intensity rating (PROP 3.2 mM), and fungiform papilla density and diameter. Main effects ANOVA was used to assess the first-order (non-interactive) effects of multiple categorical independent variables.

One-way ANOVA was used to compare the SD of diameter of fungiform papillae and the percentage of distorted fungiform papillae across gustin gene genotypes, and the effect of treatments on cell metabolic activity. Post-hoc comparisons were conducted with the Newman-Keuls test.

Stepwise, multiple linear regression was used to predict PROP phenotype (threshold and bitterness intensity rating), fungiform papilla density and morphology using gustin and *TAS2R38* genotypes, gender and age as predictor variables. The relative contribution of each significant variable and semipartial correlations (*sr*) for each variable are reported in the tables. Cell growth (expressed as percentage of control values) was compared between cells treated with saliva from individuals with genotype AA and GG of the gustin gene using the Student’s *t*-test. Statistical analyses were conducted using STATISTICA for WINDOWS (version 7.0; StatSoft Inc, Tulsa, OK, USA). *p*-values <0.05 were considered significant.

## Results

The Markov Chain test showed that the population meets the Hardy Weinberg equilibrium both for *TAS2R38* and gustin gene (*p*=0.6154 and *p*=0.1174, respectively). The distribution of the *TAS2R38* and gustin gene genotype associations is shown in Table 1. Markov Chain algorithm showed that the two loci were not in linkage disequilibrium (p=0.1782). Chi square test showed that carriers of the taster form of *TAS2R38* were not more likely to have the functional variant of the gustin gene in either the additive (χ^2^=6.5; *p*=0.17) or the dominant model (χ^2^=2.54; *p*=0.11).

**Table 1:** Number of occurrences of each combination of the *TAS2R38* and gustin gene genotypes in a genetically homogeneous cohort.

Genotype	Subjects (n)
AVI/AVI - GG	5
AVI/AVI - AG	6
AVI/AVI - AA	9
PAV/AVI - GG	1
PAV/AVI - AG	12
PAV/AVI - AA	20
PAV/PAV - GG	2
PAV/PAV - AG	2
PAV/PAV – AA	6

### PROP Thresholds and Bitterness Intensity

Molecular analysis for polymorphism *rs2274333* (A/G) of the gustin (CA6) gene allowed us to identify the genotype of sixty-three subjects: 35 were homozygous AA, 20 were heterozygous and 8 were homozygous GG. The analysis at the three SNPs of the *TAS2R38* locus identified 10 subjects who were PAV homozygous, 33 were heterozygous and 20 were AVI homozygous.

**Figure 1:**
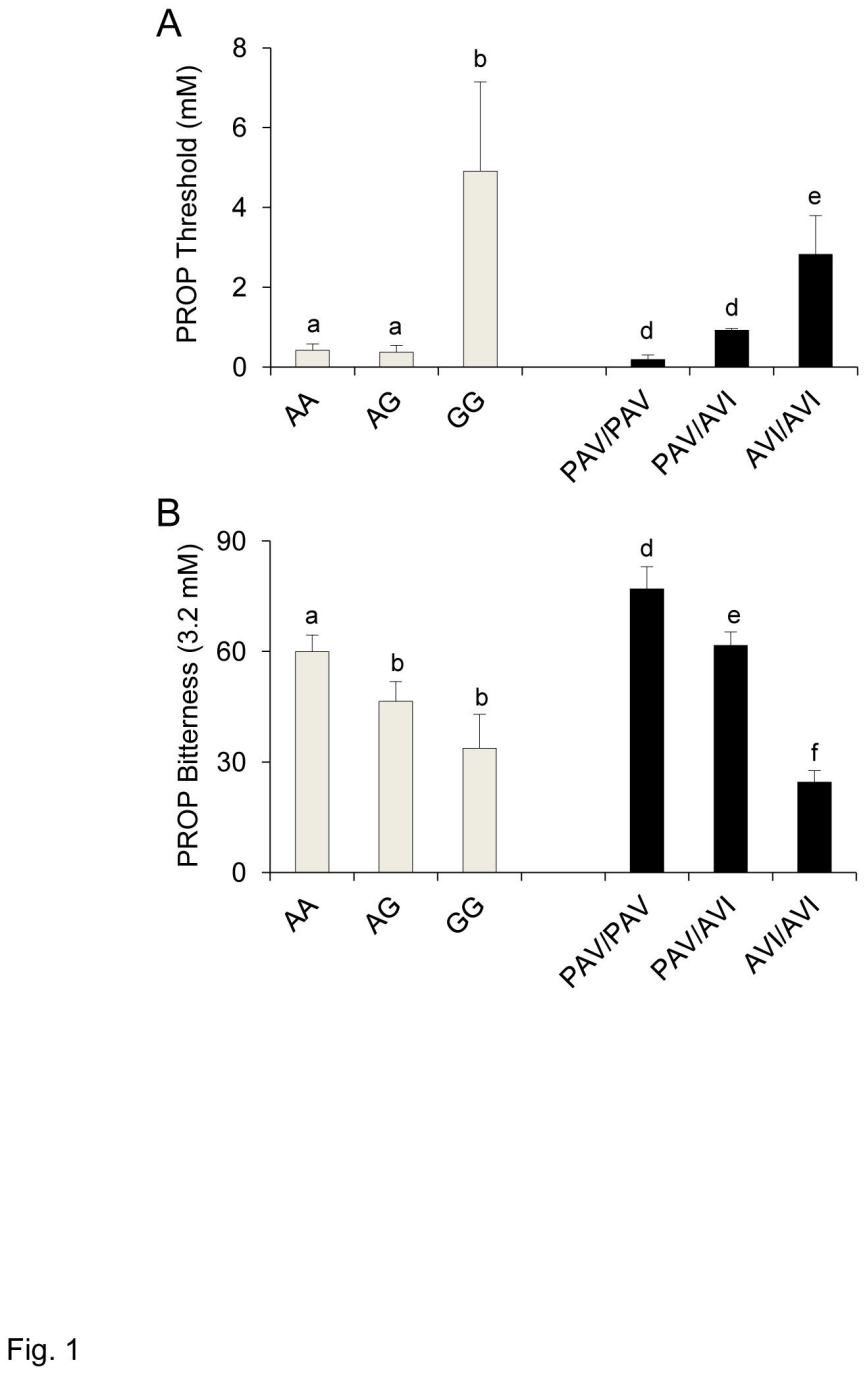
Relationship between PROP phenotype and gustin gene and *TAS2R38* polymorphisms. PROP threshold (A) and bitterness intensity ratings (3.2 mM) (B) of individuals with genotypes AA, AG and GG of gustin (CA6) polymorphism *rs2274333* (A/G), and of individuals with genotypes PAV/PAV, PAV/AVI and AVI/AVI of *TAS2R38*. All values are mean (± SEM). n=63. Different letters indicate significant difference (*p*≤0.0471; Newman-Keuls test subsequent to main effects ANOVA).

PROP threshold values and bitterness intensity ratings (PROP 3.2 mM) of individuals with genotypes AA, AG and GG of the gustin gene and with genotypes PAV/PAV, PAV/AVI and AVI/AVI of *TAS2R38* are shown in Figure 1A and B. Main effects ANOVA revealed a strong association between PROP threshold and the gustin gene polymorphism (*F*_[2,58]_ = 10.502; *p*=0.00013). Post-hoc comparisons showed that thresholds were statistically higher in individuals with genotype GG of the gustin gene than in the other genotypes (*p*≤0.000119; Newman-Keuls test), but not different between AA and AG individuals (*p*>0.05). Although thresholds were variable in those with the GG genotype, thresholds were more than 10-fold higher in these individuals than in the other groups. Main effects ANOVA also showed an association between PROP threshold and *TAS2R38* genotypes (*F*_[2,58]_ = 6.0189; *p*=0.0042). Thresholds of individuals with the AVI/AVI genotype were higher than those of individuals with genotypes PAV/PAV and PAV/AVI (*p*≤0.00158; Newman-Keuls test), that did not differ from each other (*p*>0.05).

PROP bitterness intensity ratings (3.2 mM) were strongly associated with *TAS2R38* genotypes (*F*_[2,58]_ = 32.468; *p*<0.000001) and less so with the gustin gene polymorphism (*F*_[2,58]_ = 3.4330; *p*=0.038). *TAS2R38* bitterness ratings of PAV/PAV individuals were statistically higher than those of heterozygous individuals (*p*≤0.0173; Newman-Keuls test) who in turn gave higher intensity ratings to PROP than individuals with the AVI/AVI genotype (*p*=0.00011; Newman-Keuls test). In the case of gustin, post hoc comparisons showed that PROP bitterness was statistically higher in individuals with genotype AA than in those with the other genotypes (*p*≤0.0471; Newman-Keuls test), but not different between GG and AG individuals (*p*>0.05).

### Papillae Density and Morphology

**Figure 2:**
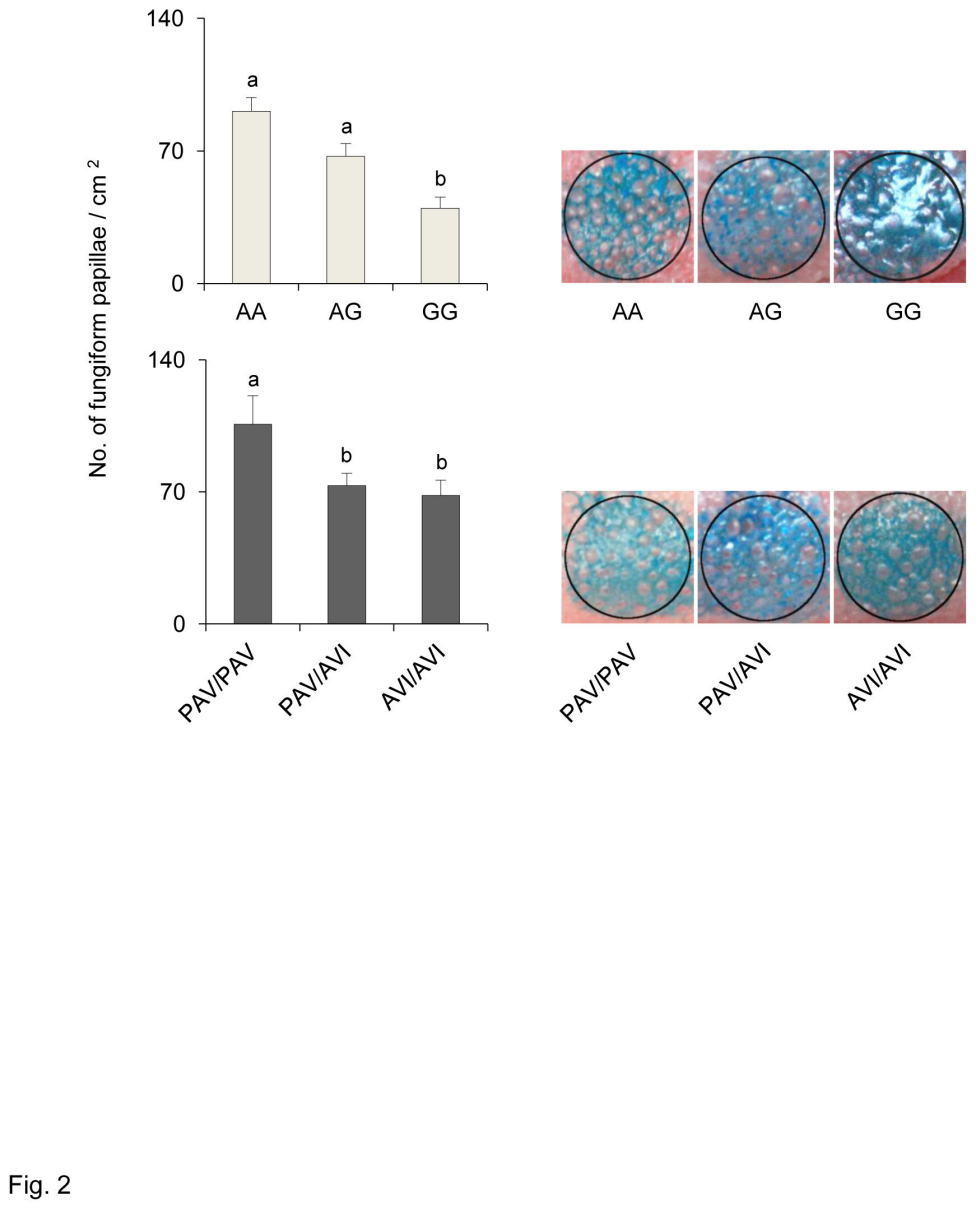
Relationship between density of fungiform papillae and gustin gene and *TAS2R38* polymorphisms. Mean values ± SEM of density of fungiform papillae (No. /cm^2^) on the anterior part of the tongue of individuals with genotypes AA, AG and GG of gustin (CA6) polymorphism rs2274333 (A/G) (upper graph) and of individuals with genotypes PAV/PAV, PAV/AVI and AVI/AVI of *TAS2R38* (lower graph). n=63. Different letters indicate significant difference (*p*≤0.0379; Newman-Keuls test subsequent to main effects ANOVA). Examples of the 6-mm-diameter stained area of the tongue tip where measures were taken are shown to the right of the graphs.

Figure 2 shows the mean densities (± SEM) of fungiform papillae on the anterior part of the tongue of individuals with genotypes AA, AG and GG of the gustin gene (upper graph) and of individuals with genotypes PAV/PAV, PAV/AVI and AVI/AVI of *TAS2R38* (lower graph). Also shown are representative images of the tongue tip stained area where measures were taken. ANOVA calculations showed that fungiform papillae density on the anterior part of the tongue was strongly associated with the gustin gene (*F*_[2,58]_ = 8.5270; *p*=0.00057) and less so with *TAS2R38* polymorphisms (*F*_[2,58]_ = 4.6147; *p*=0.0138). In the case of gustin, fungiform papillae density values were lower in individuals with the GG genotype than in those with genotypes AG and AA (*p*≤0.0379; Newman-Keuls test). Papillae density was not different between AA and AG individuals (*p*>0.05). In the case of *TAS2R38* genotypes, post hoc comparison showed that individuals with the PAV/PAV genotype had a higher fungiform papillae density than those with PAV/AVI and AVI/AVI genotypes (*p*≤0.0094; Newman-Keuls test); the density values of the latter two groups were not different from each other (*p*>0.05).

**Figure 3:**
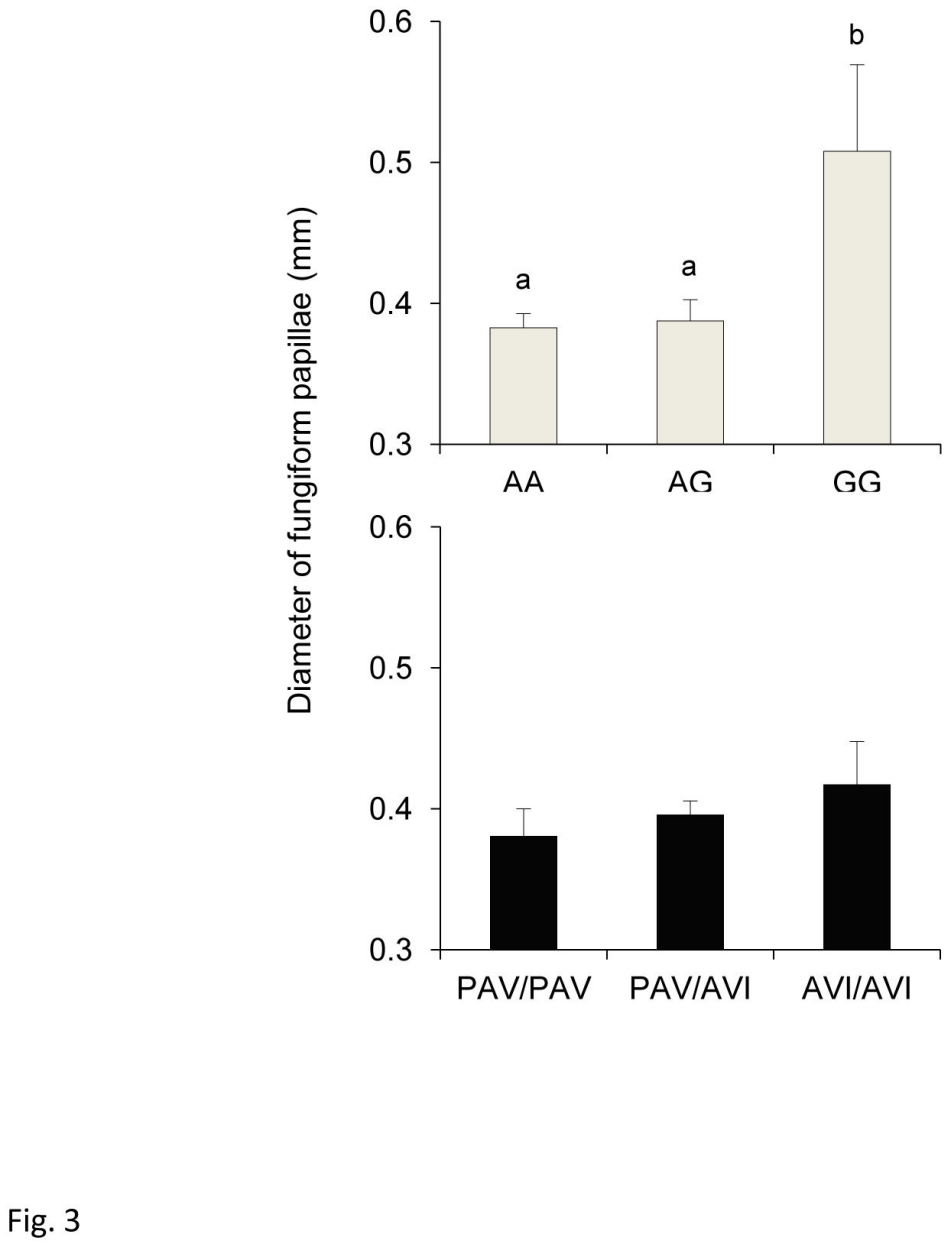
Relationship between fungiform papillae diameter and gustin gene and *TAS2R38* polymorphisms. Mean values ± SEM of the diameter of fungiform papillae of individuals with genotypes AA, AG and GG of gustin (CA6) polymorphism rs2274333 (A/G) (upper graph) and of individuals with genotypes PAV/PAV, PAV/AVI and AVI/AVI of *TAS2R38* (lower graph). n=63. Different letters indicate significant difference (*p*≤ 0.00053; Newman-Keuls test subsequent to main effects ANOVA).

ANOVA revealed that mean fungiform papilla diameter was associated with the gustin gene polymorphism (*F*_[2,58]_ = 7.5920; *p*=0.00118), but not with *TAS2R38* genotypes (*F*_[2,58]_ = 0.7191; *p*=0.491). Post-hoc comparisons showed that mean papilla diameter determined in those with genotypes AA and AG were lower than those of homozygous GG individuals (*p*≤0.00053; Newman-Keuls test) (Figure 3).

**Figure 4:**
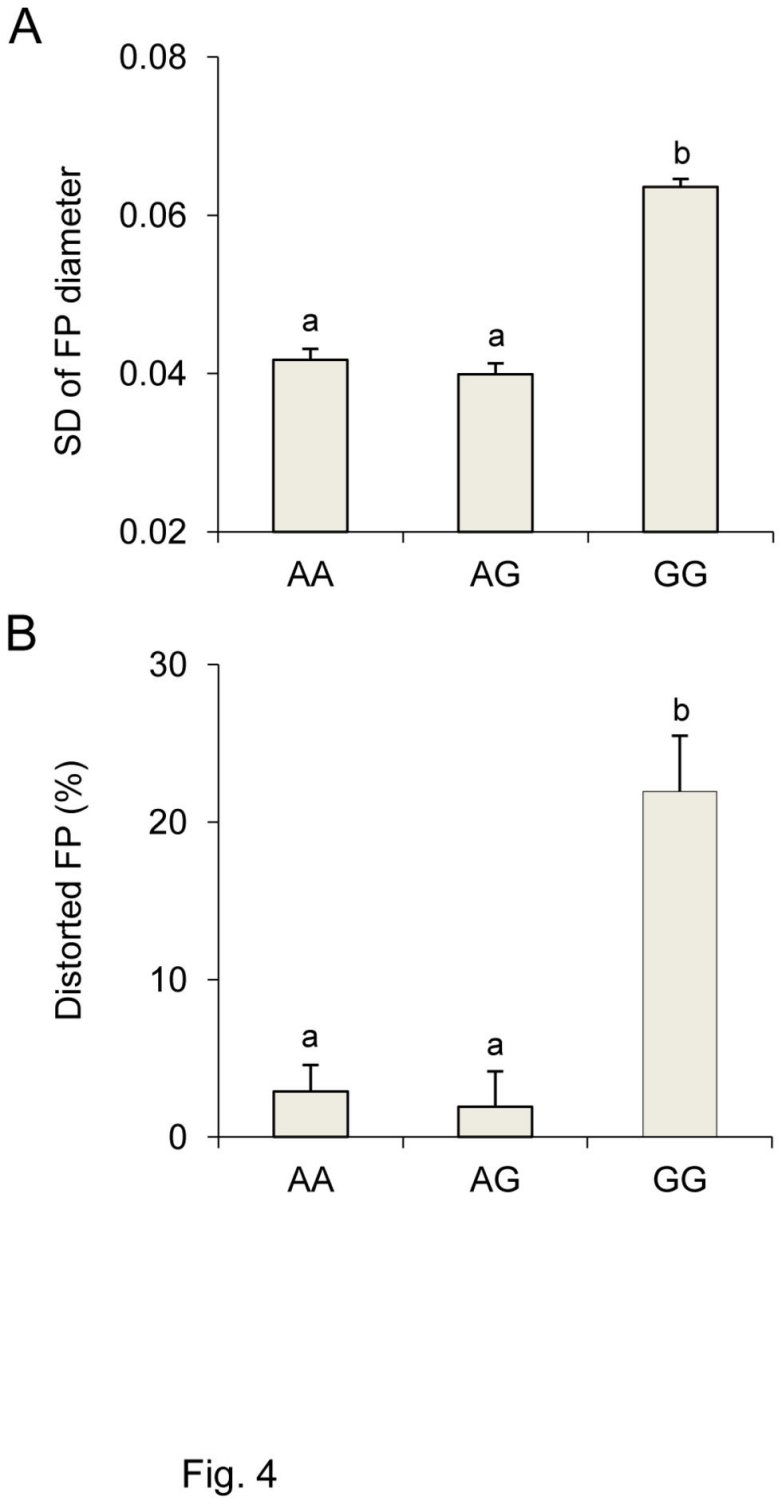
Relationship between fungiform papillae distortion and gustin gene polymorphism. Standard deviation (SD) of diameter of fungiform papillae (A) and percentage of distorted fungiform papillae (B) in individuals with genotypes AA, AG and GG of gustin (CA6) polymorphism rs2274333 (A/G). All values are mean (± SEM). n=63. Different letters indicate significant difference (*p*≤0.00019; Newman-Keuls test subsequent to one-way ANOVA).

ANOVA was also used to examine relationships between fungiform papilla morphology and gustin and *TAS2R38* genotypes. However, only associations between these features and gustin were statistically significant. In fact, both the SD of papilla diameter (Figure 4A) and the percentage of distorted papillae (Figure 4B) depended on gustin genotype (*F*_[2,60]_ = 11.765; *p*=0.00005 and *F*_[2,60]_ = 9.787; *p*=0.00021, respectively). Post-hoc comparisons showed that individuals with the GG genotype had papillae with greater variation in shape (higher SDs in papilla diameter) as well as a higher percentage of distorted papillae than the other genotypes (*p*≤0.00019 and *p*≤0.00017; Newman-Keuls test). No differences were found between AA and AG individuals (*p*>0.05).

### Multiple Regression Modeling

Multiple linear regression was used to assess the relative contributions of gustin and *TAS2R38* polymorphisms to PROP tasting and papillae density and morphology (Tables 2 and 3). Accordingly, gustin genotypes, *TAS2R38* genotypes and age were significant predictors of PROP threshold, with each factor contributing 17.72%, 11.18% and 5.45%, respectively, to the model. The overall model predicted 31.01% of the variance in threshold sensitivity. In the case of PROP bitterness intensity, *TAS2R38* and gustin genotypes were the only significant contributors in the model, predicting 55.16% of the variance in PROP bitterness intensity. However, *TAS2R38* genotype was a much stronger predictor in this model (49.75% variance) than was gustin genotype (6.18% variance).

**Table 2:** Stepwise forward multiple regression models for PROP phenotype (threshold and bitterness intensity).

PROP phenotype	Variable	Overall model		Parameter estimate			Each step
		(adj R^2^)	(*p*)	(*sr*)	(*p*)	(R^2^)	
Threshold	Gustin	0.3101	<0.001	-0.38	<0.001	0.1772	
	*TAS2R38*			-0.31	0.005	0.2890	
	Age			0.23	0.031	0.3435	
Bitterness intensity	*TAS2R38*	0.5516	<0.001	0.65	<0.001	0.4975	
	Gustin			0.26	0.004	0.5593	

Independent variables for both models included: Gustin genotypes, *TAS2R38* genotypes, age and gender. Only the significant variables are indicated. Adj, adjusted; sr, semipartial correlation.

**Table 3:** Stepwise forward multiple regression models for fungiform papilla density and morphology (diameter of papillae, SD of diameter and percentage of distorted papillae).

	Variable	Overall model		Parameter estimate			Each step
		(adj R^2^)	(*p*)	(*sr*)	(*p*)	(R^2^)	
Density of papillae	Gustin	0.3090	<0.001	0.43	<0.001	0.1952	
	Age			-0.31	0.004	0.3060	
Diameter of papillae	Gustin	0.1218	0.007	-0.36	0.004	0.1320	
SD of diamenter	Gustin	0.1342	0.005	-0.34	0.005	0.1358	
% of distorted papillae	Gustin	0.1538	0.002	-0.38	0.002	0.1611	

Independent variables for all models included: Gustin genotypes, *TAS2R38* genotypes, age and gender. Only the significant variables are indicated. Adj, adjusted; sr, semipartial correlation.

Gustin genotypes and age were the only significant contributors to fungiform papillae density with the overall model explaining 30.90% of the variance. Finally, gustin genotype was the only significant contributor to fungiform papillae diameter, SD of papilla diameter and percentage distortion. However, the predictive power of these models were relatively low, explaining 13.2-16.11% of the variance in these measures.

### In vitro experiments

**Figure 5:**
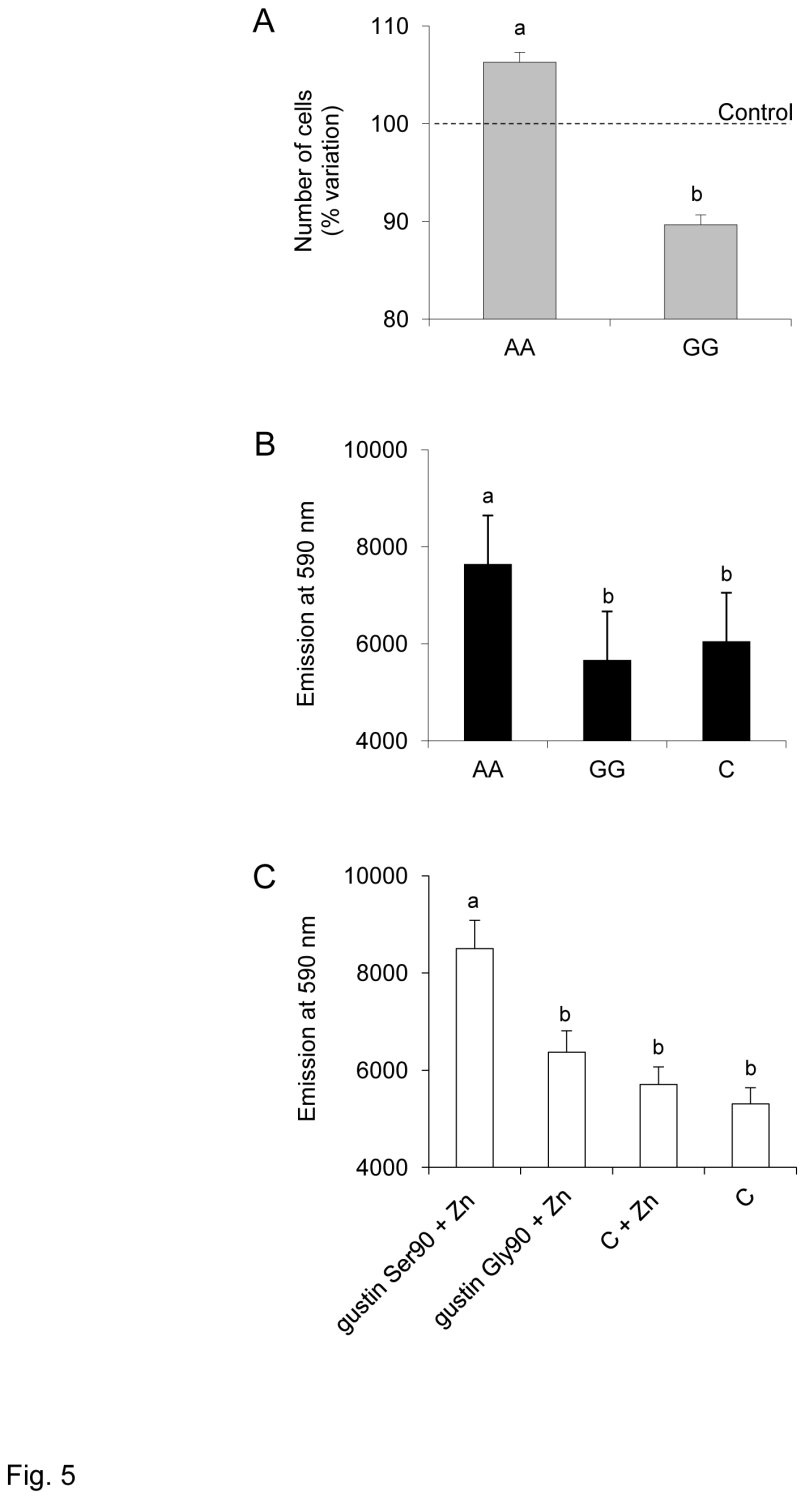
Effect of gustin gene polymorphism *rs2274333* (A/G) *in vitro* experiments. A, Number of cells, expressed as percentage of control, after treatments with saliva of subjects with genotype AA (n=12) or with saliva of subjects with genotype GG (n=12); different letters indicate significant difference (*p*=0.0135; Student’s *t* test). B, Fluorescence emission at a wavelength of 590 nm obtained from cells treated for 72 h with saliva of subjects with genotype AA, genotype GG and control; n=12; different letters indicate significant differences (*p*≤0.00023; Newman-Keuls test subsequent to one-way ANOVA). C, Fluorescence emission at a wavelength of 590 nm obtained from cells treated for 72 h with the two iso-forms of isolated gustin (gustin Ser90 or gustin Gly90) + Zn, control + Zn, or control; n=33; different letters indicate significant differences (*p*≤0.00067; Newman-Keuls test subsequent to one-way ANOVA).

The effect of gustin gene polymorphism rs2274333 (A/G) from the *in vitro* experiments is shown in Figure 5. The number of cells, expressed as a percentage of control, treated with the saliva of subjects with genotype AA (n=12) was higher than the number of cells treated with saliva of subjects with genotype GG (n=12) (*p*=0.0135; Student’s *t* test) (Figure 5A). ANOVA showed that the fluorescence emission at a wavelength of 590 nm, as a function of cell metabolic activity, depended on treatments performed with the saliva of subjects with different genotypes for the polymorphism in the gustin gene (*F*_[2,33]_ = 16.628; *p*=0.00001) (Figure 5B). Post hoc comparisons showed a higher emission of fluorescence from cells treated with saliva of subjects with genotype AA than that obtained from cells treated with saliva of genotypes GG (*p*=0.000137; Newman-Keuls test) or control (*p*=0.000229; Newman-Keuls test). No differences were found between treatment with saliva of genotypes GG and control (*p*>0.05).

ANOVA also showed that the fluorescence emission depended on treatments performed with the two iso-forms of gustin (gustin Ser90 or gustin Gly90) (*F*_[3,128]_ = 10.463; *P* < 0.00001) (Figure 5C). Pairwise comparisons showed that cells treated with gustin 90Ser + Zn emitted a higher fluorescence than those treated with gustin 90Gly + Zn or with control + Zn or control (*P* ≤ 0.0067; Newman-Keuls test). No differences were found between these last three treatments *P* > 0.05.

## Discussion

One aim of the present study was to determine the effects of *TAS2R38* genotypes and the rs2274333(A/G) polymorphism in the gustin gene on PROP tasting, fungiform papillae density and morphology. Results showed that PROP thresholds and bitterness intensity ratings were associated with *TAS2R38* and gustin gene genotypes, as reported previously [49]. Importantly, those who were homozygous GG for the gustin SNP had thresholds that were more than 10-fold higher than those who carried either the AA or AG forms suggesting that gustin has a fundamental role in the ability to taste PROP at low concentration. Both gustin and *TAS2R38* genotypes were associated with fungiform papillae density with a stronger effect for gustin than for *TAS2R38*. However, only gustin was associated with morphological changes in fungiform papillae such as larger size, greater variation in shape and more distortions.

Regression modelling permitted us to assess the relative contributions of gustin and *TAS2R38* genotypes to these same outcomes. Both genes contributed to threshold acuity, however, *TAS2R38* polymorphisms made a much greater contribution to PROP bitterness intensity than did gustin. These data confirm the findings of Calò et al. [49] showing a much stronger effect of *TAS2R38* genotypes on suprathreshold intensity than threshold sensitivity. The reasons for these differential effects are unclear, but we can speculate that at low stimulus concentrations, that are further diluted in the oral cavity, both papillae features (as determined by gustin) and the presence of the functional, PAV form of the TAS2R38 receptor are critical for tasting PROP. At higher concentrations, when there is a higher probability that the stimulus molecules arrive at the receptor site, the number of functional (PAV) receptors may be more important for enhancing peripheral nerve signalling than the number of taste cells that are present. This explanation may be overly simplistic as it fails to account for a number of factors that affect taste function such as smoking, damage to taste nerves [71,72] and variability in TAS2R38 receptor expression. These factors need to be considered in future studies to obtain a more complete picture of the physiological mechanisms contributing to PROP tasting.

Our data showed that *TAS2R38* genotypes were associated with papillae number, and PAV homozygous individuals had a higher papillae number with respect to other genotypes. However, in the regression analysis, that looks at multiple variables at the same time, the *TAS2R38* genotypes were not significant predictors of papillae number or their other morphological features. It is important to note however, that gustin genotypes predicted only a small percentage of the variance in papillae size, and shape, suggesting that other factors define these morphological characteristics. We did not investigate brain-derived neurotrophic factor (BDNF) which has also been implicated in papillae development and maintenance [73–75], and this also needs to be pursued in future investigations.

Numerous studies have report greater papillae densities in PROP super-tasters compared to those who perceive PROP as less intense [2,34,54–56,76]. In agreement with these studies we found that homozygous individuals for the sensitive allele (PAV) of *TAS2R38*, who perceived the highest PROP bitterness, had higher papillae densities compared to those who perceived PROP as less intense. Our results complement these earlier observations by also showing that a single A allele in the gustin gene was sufficient to increase papillae density. In addition, we studied for the first time, the relationship between papillae distortion, which seems to be a measure of functionality [53], and genotypes for the two loci. We found that a single A allele in the gustin gene produced small papillae with a regular morphology; these effects were not found for *TAS2R38* genotypes.

Hayes et al. [35] reported no association between *TAS2R38* genotypes and papillae densities. In our previous work [49] we found that *TAS2R38* and the gustin gene had independent effects in modulating PROP phenotype in an ethnically homogeneous population where the majority of PAV homozygotes also carried the AA (functional) form of the gustin *rs24743333* polymorphism. In contrast, a majority (55%) of AVI homozygotes carried the GG (less functional) form. In the present study, fewer AVI homozygotes (25%) carried the GG form. Nevertheless, the presence of the AA form of gustin was more common in those with at least one PAV allele for *TAS2R38*. Thus, it is plausible that the higher papillae densities we observed in PAV homozygotes (although the sample size for this group was low) may better reflect the actions of gustin rather than *TAS2R38* genotypes. Future studies will have to confirm this finding. Our results should not lead to the conclusion that *TAS2R38* genotypes predict gustin genotypes. The two loci are independent (not in linkage disequilibrium) and, in fact, reside on different chromosomes. Why these two discrete loci appear to have functional overlap in defining PROP tasting and papillae density and morphology is presently unknown. The answer to this question cannot be resolved here and will come from more comprehensive genetic studies.

Up to now, only few populations have been tested for variants in the gustin gene, but the allele frequencies in these populations are not known. Variations in the frequency of gustin A and G alleles across populations could produce discrepant findings across studies, and could explain why a genome wide phenotype-genotype association study of PROP threshold failed to detect a relationship with variants in the gustin gene [5]. Both confounding and heterogeneity of populations are common contributors to the problem of non replication in genetic studies of complex traits [77]. On the other hand, the study of ethnically homogeneous populations can be expected to reduce noise in genetic association studies by diminishing ancestral diversity [77–79]. The genetic homogeneity of the population we studied might have allowed us to observe the effect of the gustin gene as growth factor of taste buds. We also found that in regression analysis, *TAS2R38* accounted for less variance in the threshold response to PROP than in previous studies [8,35,80]. This finding could also reflect underlying differences in population characteristics.

For more than 40 years, gustin has been described as a trophic factor responsible for the growth and maintenance of taste buds [50]. This role was based on observations of patients with taste loss who exhibited pathological changes in taste buds accompanied by low salivary gustin and zinc levels. Administration of zinc to a subset of these patients improved taste function, increased salivary gustin and normalized taste bud morphology [53]. However, direct evidence that gustin increases cell growth has been lacking. Our *in vivo* studies showed that treatment of cells with saliva from individuals with the AA genotype of gustin resulted in increased cell proliferation and metabolic activity, whereas similar treatment with saliva from individuals with the GG genotype did not. Furthermore, direct treatment of cells with the active iso-form of the protein (gustin90Ser) increased cellular metabolic activity, while treatment with the inactive iso-form (gustin 90 Gly) failed to do so. These novel findings confirm, for the first time, a role for gustin in cell proliferation and maintenance.

In conclusion, our findings in an genetically homogeneous cohort suggest that the gustin (CA6) gene polymorphism, *rs2274333* (A/G), affects PROP tasting by acting on the density and maintenance of fungiform papillae, and that between the two protein iso-forms that result from this polymorphism, gustin 90Ser exhibits full functional activity, compared to the gustin 90Gly iso-form. In addition, the results of this work, if confirmed in different populations, will provide a mechanistic explanation of why PROP super-taster individuals have a higher density of fungiform papillae than PROP non-tasters, and why they show greater oral responsiveness to a wide range of stimuli that are not mediated via the TAS2R38 bitter taste receptor.
